# Fatigue Performance of Ti-6Al-4V Additively Manufactured Specimens with Integrated Capillaries of an Embedded Structural Health Monitoring System

**DOI:** 10.3390/ma10090993

**Published:** 2017-08-25

**Authors:** Michaël Hinderdael, Maria Strantza, Dieter De Baere, Wim Devesse, Iris De Graeve, Herman Terryn, Patrick Guillaume

**Affiliations:** 1 Department of Mechanical Engineering, Vrije Universiteit Brussel, 1050 Brussels, Belgium; dieter.de.baere@vub.be (D.D.B.); wim.devesse@vub.be (W.D.); patrick.guillaume@vub.be (P.G.); 2 Department of Mechanics of Materials and Constructions, Vrije Universiteit Brussel, 1050 Brussels, Belgium; maria.strantza@vub.ac.be; 3 Department of Electrochemical and Surface Engineering, Vrije Universiteit Brussel, 1050 Brussels, Belgium; iris.de.graeve@vub.be (I.D.G.); herman.terryn@vub.be (H.T.)

**Keywords:** structural health monitoring, fatigue, Ti-6Al-4V, additive manufacturing, 3D printing, laser metal deposition, directed energy deposition, eSHM

## Abstract

Additive manufacturing (AM) of metals offers new possibilities for the production of complex structures. Up to now, investigations on the mechanical response of AM metallic parts show a significant spread and unexpected failures cannot be excluded. In this work, we focus on the detection of fatigue cracks through the integration of a Structural Health Monitoring (SHM) system in Ti-6Al-4V specimens. The working principle of the presented system is based on the integration of small capillaries that are capable of detecting fatigue cracks. Four-point bending fatigue tests have been performed on Ti-6Al-4V specimens with integrated capillaries and compared to the reference specimenswithout capillaries. Specimens were produced by conventional subtractive manufacturing of wrought material and AM, using the laser based Directed Energy Deposition (DED) process. In this study, we investigated the effect of the presence of the capillary on the fatigue strength and fatigue initiation location. Finite element (FEM) simulations were performed to validate the experimental test results. The presence of a drilled capillary in the specimens did not alter the fatigue initiation location. However, the laser based DED production process introduced roughness on the capillary surface that altered the fatigue initiation location to the capillary surface. The fatigue performance was greatly reduced when considering a printed capillary. It is concluded that the surface quality of the integrated capillary is of primary importance in order not to influence the structural integrity of the component to be monitored.

## 1. Introduction

Instead of removing material from an existing block of material, additive manufacturing (AM) technologies form a component by fusing material in a layerwise manner. The three-dimensional design is split into different layers, each layer containing two-dimensional cross sectional information of the component at a particular height in the component. Starting from the base plate, material is selectively added according to the two-dimensional shape defined in the layer. Once a layer is completed, the next layer is added on top of the previous one until the build is completed. AM was initially introduced as being a prototyping technology. Only recently has AM been considered a promising technique for functional part production [1]. The design freedom offered by AM is a key enabler for weight optimization through topology optimization while allowing to introduce functions inside the component. Near net shape production of functional parts reduces material waste, material cost and lead times since forged or cast base material are no longer necessary [2]. As a result, industrial sectors (e.g., aerospace) are interested in the AM technology for the production of functional parts.

The high quality standards requested by the aerospace industry demand a good understanding of the material properties. Functional parts are challenged to survive the component’s intended service life. Therefore, fatigue properties of AM components are widely studied nowadays. Two main metal AM process categories can be distinguished. Powder Bed Fusion (PBF) processes, on the one hand, are AM processes in which thermal energy selectively fuses regions of a powder bed. The most famous examples are Selective Laser Melting (SLM) and Electron Beam Melting (EBM) in which, respectively, a laser and electron beam are focused to fuse the particles of the powder bed. On the other hand, Directed Energy Deposition (DED) is an AM process in which thermal energy is used to fuse materials by melting powder particles as they are being deposited. Laser Metal Deposition (LMD), laser cladding or Laser Engineered Net Shaping (LENS) are some common trade names that are often used to refer to the laser based DED process in which a laser beam is used to melt the injected powder particles. The Ti-6Al-4V material is one of the main AM materials that is widely investigated for aerospace applications. The mechanical response of the AM Ti-6Al-4V is related to the AM process that is used. Chastand et al. [3] investigated the fatigue performance of Ti-6Al-4V produced by laser based PBF in both High Cycle Fatigue (HCF) and Low Cycle Fatigue (LCF) conditions. The authors found that the high roughness of as-built specimens involved a high amount of surface defects that were critical for fatigue performance. Process inherent defects have a major influence on the fatigue performance of Ti-6Al-4V produced by laser based PBF [4]. Regardless of the applied load ratio, it was found that all specimens failed due to crack initiation at defects originating from the production process. Defects play a critical role in laser based PBF Ti-6Al-4V fatigue performance. As-built surfaces become crack initiation sites, especially when the maximum tensile stress is higher than 500 MPa, as reported by Gong et al. [5]. Many more works report that fatigue performance of Ti-6Al-4V samples produced by laser based PBF are negatively affected by surface roughness, pores, residual stresses and internal defects [6,7,8]. The work of Greitemeier et al. [2] compared the fatigue performance of Ti-6Al-4V specimen produced by laser based PBF and electron beam based PBF. The authors concluded that the lower fatigue limits of the specimens produced by electron beam based PBF can be ascribed to the higher surface roughness of the specimens produced by electron beam based PBF as compared to laser based PBF. Hrabe et al. [9] investigated the fatigue properties of Ti-6Al-4V produced by electron beam based PBF and found that voids, due to localized incomplete melting, were present at all crack initiation sites. The fatigue strength at $10^{7}$ cycles (200–250 MPa) was significantly lower than the ones reported in a similar study (400–450 MPa) [10]. It was hypothesized that fatigue performance was greatly reduced due to a higher prevalence and severity of voids. Edwards et al. concluded from his research that the fatigue performance of Ti-6Al-4V specimens produced by electron beam based PBF was significantly lower than that of wrought material due to defects such as porosity and surface roughness [11]. Significant increases in fatigue resistance can be achieved by combining HIP and thermal treatments [12]. The fatigue performance of Ti-6Al-4V produced by laser based DED has also been discussed in the literature. In the work of Sterling et al. [13], fatigue lives of Ti-6Al-4V produced by laser based DED were found to be shorter than wrought Ti-6Al-4V, largely due to pores. The fatigue life of Ti-6Al-4V produced by laser based DED is predominately controlled by the presence of physical defects, in particular the unmelted particles at the surface [14].

Although the high number of process parameters involved in an AM process has led to widespread fatigue performances, most of them report significant reduction of fatigue life due to the presence of voids, unmelted regions and increased surface roughness. Currently, the lack of proper AM process monitoring and control results in unrepeatable material behaviour. Given that Structural Health Monitoring (SHM) is used to provide continuous information about the structural integrity of a component, the implementation of such a system on AM components is helpful to detect early deterioration of the component. Up to now, a large amount of small sensors of multiple types are necessary to deploy SHM systems over large areas and moving the technology beyond hot-spot monitoring [15]. In an attempt to reduce the amount of sensors used, to reduce their installation cost and reduce the environmental impact (noise, accidental damage, hazardous conditions, etc.) on the SHM system performance, integrated SHM systems are currently under investigation [15,16,17].

In this work, the fatigue behaviour of Ti-6Al-4V produced by laser based DED with an integrated SHM system will be discussed. The working principle of the ’effective Structural Health Monitoring’ system (eSHM) is based on the integration of small capillaries inside the structure. The capillaries are initially pressurized and a pressure sensor continuously interrogates the static pressure level inside the capillaries. The leak flow that results from a fatigue crack breaching the capillary will change the capillary pressure level towards ambient conditions. As such, fatigue cracks can be detected using the eSHM system [18,19]. In the work of Strantza et al. [20], the reliability of the crack detection capability of the eSHM system was proven. The latter work used different Non Destructive Testing (NDT) techniques in order to confirm the existence of the crack, thereby coping with the issue of false positive detections. Furthermore, no false negatives were reported as all fatigue tests were succesfully stopped by the eSHM prior to the final failure, which proves that the eSHM system reached technology readiness level (TRL) 3 [21]. Further tests on Ti-6Al-4V specimens with the integrated eSHM system produced by laser based PBF showed that the integration of the eSHM had no influence on the crack initiation location. Fractographic analysis revealed that all cracks initiated from near-surface defects such as concentrated pores or lack-of-fusion regions [22].

The current work extends the latter work [22] to Ti-6Al-4V specimens produced by laser based DED, another common AM process. Although dimensionally less accurate, this technique offers some advantages over laser based PBF such as higher build speeds and addition on existing parts (e.g., repairs). The fatigue performance of Ti-6Al-4V specimenswith the integrated capillaries were evaluated. The impact on the fatigue performance of using AM technologies to integrate a capillary inside Ti-6Al-4V specimens is dual. On the one side, as reported in the literature, the use of AM technologies itself alters the fatigue performance. On the other side, the presence of a capillary might also affect the fatigue performance of the Ti-6Al-4V specimens. In order to separately analyse the effect of using AM technologies and the introduction of the capillary of the eSHM system, the fatigue performance of specimens produced from wrought material (with and without capillary) were compared to those of additively manufactured specimens (with and without capillary). Seven Ti-6Al-4V specimens, produced by laser based DED, were tested during four-point bending fatigue tests. Five of them having an integrated capillary of the eSHM system while two of them have no capillary. Six more specimens were produced from wrought Ti-6Al-4V of which three have an integrated capillary and three other specimens have no capillaries. The capillary surface roughness, hardness and fatigue strengths were compared and a fractographic analysis using Scanning Electron Micrography (SEM) images has pinpointed the fatigue initiation location. The conclusions drawn from these comparisons were verified using finite element (FEM) simulations.

## 2. Materials and Production

**Figure 1:**
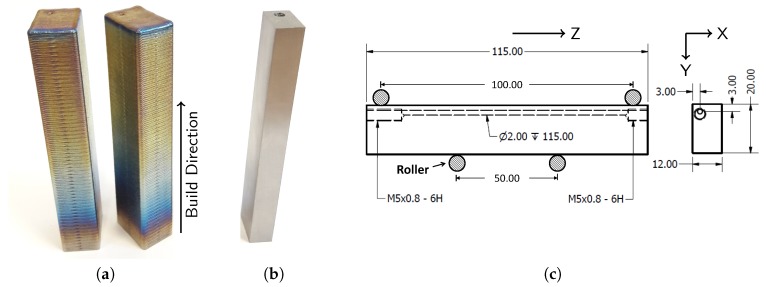
Additive manufactured Ti-6Al-4V specimen produced by laser based DED in as-built condition (**a**) before milling and (**b**) after milling according to the design presented in (**c**).

The wrought specimens were produced by conventional milling operations from a mill annealed Ti-6Al-4V plate. The capillaries were added according to the design presented in Figure 1c, using deep gun drilling with a drill diameter of 2 mm.

The additively manufactured specimens were produced by laser based DED using a 7 kW IPG YLS-7000-S2 fibre laser (IPG Photonics, Oxford, MA, USA) with a diameter of 600 µm. The laser beam passed through a focal lens and collimator, which resulted in a laser spot diameter of 1200 µm on the substrate. The specimens were built at a constant laser power of 500 W while the nozzle was moving at a linear scan speed of 1000 mm/min. The layer thickness was 500 µm. The powder was transported through a continuous coaxial nozzle (Fraunhofer—Institüt für Lasertechnik, Munich, Germany) using argon as transport and shielding gas. The transport gas flow rate varied between 6–8 L/min blowing 2.96 g/min Ti-6Al-4V particles into the melt pool. Specimens were built on Ti-6Al-4V flat plates with a thickness of 18 mm. The build direction was vertical (indicated on Figure 1a) and the scanning direction of successive layers was rotated by an angle of 90${}^{\circ }$. In one layer, the contours were first scanned before a bidirectional scanning pattern with a scan offset of 0.3 mm was applied. Figure 1a shows two laser based DED samples in as-built condition. AM processes, such as laser based DED, can introduce high thermal stresses inside the specimen. After the specimens were removed from the base plate, a heat treatment was applied in order to obtain stress relieved conditions. While additively manufactured Specimens 11–13 were subjected to a standard heat treatment often used for conventional Ti-6Al-4V (4 h at 650 ${}^{\circ }$C), Specimens 7–10 were subjected to a heat treatment at lower temperatures in order not to alter the microstructure (2 h at 530 ${}^{\circ }$C). The specimens were then milled to the final dimensions of 115 mm × 20 mm × 12 mm. The corners of the specimens were not rounded. Although the capillaries were designed to have a diameter of 2 mm, the printed capillaries in Specimens 9–13 turned out to have variable diameters varying between 1.4–1.8 mm due to the geometrical inaccuracy of the laser based DED process. In order to improve the capillary surface roughness, the printed capillaries of Specimens 11–13 were drilled through using deep gun drilling with a drill of 2 mm diameter.

The location of the capillary is crucial in terms of fatigue crack detection by the eSHM system and fatigue performance of the specimen. A trade-off is to be made. A closer location of the capillary near the tensile stressed surface of the specimen allows earlier detection of growing cracks, but increases the chances of crack initiation at the capillary surface. A capillary topology study was performed by Strantza et al. on specimens produced by laser based DED. It was found that, for both capillaries with their center located at 3 mm and 4 mm from the bottom and side of the specimen, the crack initiated at imperfections on the surface of the printed capillary. It was concluded first that other parameters such as residual stresses and capillary surface roughness have to be eliminated before detailed capillary topology studies can be started [23]. The current work therefore investigates the effect of the capillary surface roughness in stress relieved specimens. Corresponding to the designs used in the work of Strantza et al. [23], the capillaries were positioned such that their center was located at 3 mm from both the side and bottom of the specimen, as shown in Figure 1c. As such, one corner of the specimen is more influenced by the presence of the capillary than the other. Two M5 threaded connections were then foreseen at the ends of the straight capillary. Figure 1b shows a specimen with capillaries after milling. The specimens with integrated capillaries of the eSHM were all produced according to the design presented in Figure 1c.

## 3. Experimental Study

### 3.1. Installation of the eSHM System

**Figure 2:**
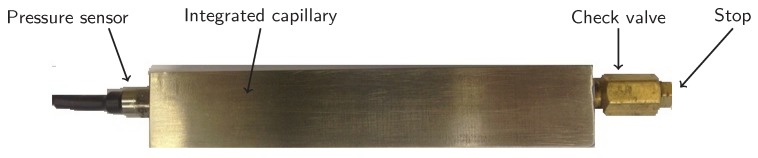
Fully equipped Ti-6Al-4V specimen with installed pressure sensor at one side and check valve and stop at the other side. The eSHM system is active.

The specimens were equiped with a Kulite XTL-123BEG-190M-1.7 bara pressure sensor (Kulite Semiconductor Products Inc., Leonia, NJ, USA) at one side and a Clippard MCV-1-M5 check valve (Clippard Instrument Laboratory Inc., Cincinnati, OH, USA) on the other side. In order to properly seal the connections, a Loctite 577 thread sealant (Henkel, Düsseldorf, Germany) was applied to the M5 threads. The anaerobic thread sealant remains liquid until being isolated from oxygen in the presence of the metal ions of the thread. When cured, they form a durable seal by filling up the gaps between the threads. The specimen were then placed in a vacuum oven and the pressure inside the capillary was reduced to approximately 0.4 bara. The check valve prevented the capillary pressure to rise when taken out from the vacuum oven. In order to prevent occasional leaking through the check valve, an additional Clippard 11755-M5-PKG stop (Clippard Instrument Laboratory Inc., Cincinnati, OH, USA) was screwed onto the check valve end. Figure 2 shows the Ti-6Al-4V specimen with the installed eSHM system, just before testing.

### 3.2. Test Procedure

**Figure 3:**
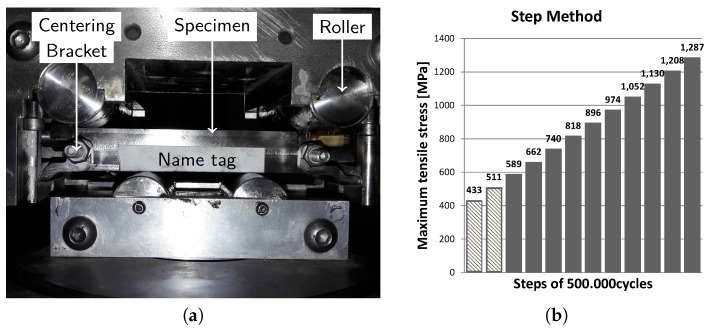
All specimens were subjected to four-point bending fatigue tests according to the step method. (**a**) specimen installed in the four-point bending test setup and (**b**) the maximum tensile stress levels in each load step.

All specimens were subjected to a cyclic fatigue loading in a four-point bending test setup. Figure 3a shows the specimen installed in the test bench. In between the two inner rollers, a region of maximum and constant stresses is present in the specimen. The fatigue cracks are expected to nucleate and grow in this region, on the tension side of the specimen. The specimen was placed in the set-up with the capillary in the tensional stress area, such that the eSHM system could detect the fatigue cracks as early as possible. The centering brackets held the specimen in place during testing.

As suggested from the literature [24,25,26,27], the step-method is considered as a fast methodology to estimate the fatigue strength of a specimen. The experimental observations presented in Nicholas et al. [24] indicate that the step-loading procedure used in their investigation provides a valid method for determining the fatigue limit stress corresponding to a given number of cycles in the high cycle regime. According to the step method, a large number of cycles (considered to be run-out) at constant load level are applied to the specimen. After each completed step, the load level is increased with a fraction of the initial load level while keeping the stress ratio constant. This procedure is generally repeated until failure of the specimen.

In this particular study, each step consisted of 500,000 cycles of a sinusoidal load with a stress ratio (R ratio) equal to 0.1 and a testing frequency of 15 Hz. The initial load level must be considered below the expected fatigue strenght of the specimen. From the literature [22], indications of the failure level of wrought Ti-6Al-4V under the same testing configurations were found to be equal to 820 MPa. The initial stress level was chosen to be 589 MPa. Then, every step, the stress level in the section with maximum stress levels was increased by approximately 76 MPa such that failure of the reference samples is expected in the fourth step. However, when a specimen failed in the first step, all specimens with the same configuration were started at a lower initial stress level with a minimum of 433 MPa. The different loading steps during the four-point bending tests are depicted in Figure 3b. The initial stress levels that were used can be found in the summarization Table 1.

### 3.3. Test End Definition

The test procedure described in the previous paragraphs is generally repeated until failure of the specimen. During this investigation, the failure of a specimen was defined differently for specimens with and without embedded eSHM system. Specimens without integrated eSHM system were subjected to the loading until the deformation of the specimen exceeded pre-set limits corresponding to the complete rupture of the specimen.

**Figure 4:**
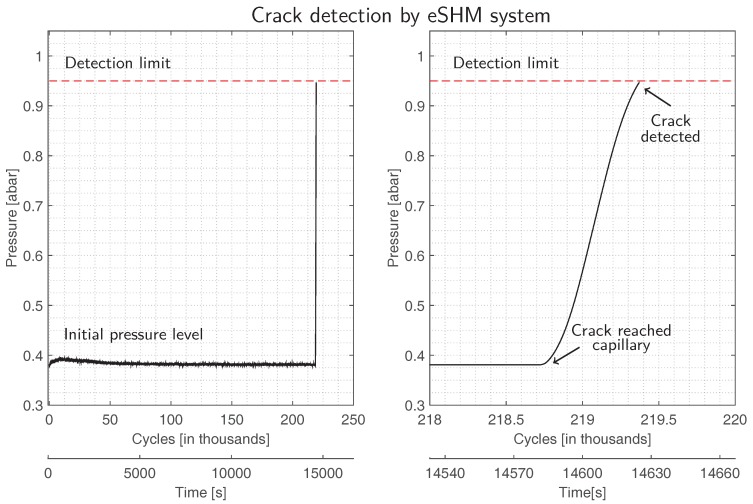
Crack detection by eSHM system for Specimen 13. Capillary pressure output during the entire test (**left**) and a detailed view on crack detection (**right**).

On the contrary, the test procedure of specimens with integrated eSHM systems was stopped at crack detection by the eSHM system. Figure 4 shows the pressure output of the eSHM system during the entire test (left) and a detailed view on the last cycles of the test (right). Initially put under vacuum conditions, the capillary became very sensitive to leaks. During initial testing, no crack was present in the test specimen and the internal capillary pressure remained unchanged and equal to the initial pressure level of approximately 0.4 bara (see Figure 4 (left)). However, a fatigue crack initiated at the specimen’s outer surface and grew inward towards the capillary. When the crack finally breached the capillary, the crack formed a leak connection between the capillary and the ambient conditions outside the specimen. The resulting leak flow increases the pressure level inside the capillary towards theambient pressure level. According to Figure 4 (right), this happened after about 218,750 cycles or 14,585 s of testing for Specimen 13. In the following period, the pressure sensor installed at the capillary end registers a rapid pressure increase towards ambient pressure conditions. The test procedure was stopped when the capillary pressure exceeded the pre-set limit of 0.95 bara. Figure 4 (right) indicates that, according to this methodology, Specimen 13 failed after 219,377 cycles. All test specimens were successfully stopped by the eSHM system. The specimen still withstood the loading and was not ruptured. In order to be able to inspect the fracture surface, the specimens were loaded further at the load level of the last step until final rupture of the specimen occurred.

### 3.4. Analysis of the Test Results

Besides a fractographic analysis to retrieve the fatigue initiation location, some additional tests were performed to provide more insight in the fatigue test results. As fatigue initiation is strongly depending on the surface finish of the specimens, the surface roughness of the tensile stressed surface of the specimens was determined on a Perthometer PRK (Mahr GmbH, Göttingen, Germany). With a scan length of $l_{t}$ = 4.8 mm and a cut-off wavelength of 0.8 mm, four different scans were performed near the location of the fracture (two on each side of the fracture). The average $R_{a}$ and maximum $R_{t}$ values are reported in Table 1. All specimens had an external surface roughness of $0.24\;\mu m\;\leq \;R_{a}\;\leq \;0.79\;\mu m$ and $2.59\;\mu m\;\leq \;R_{t}\;\leq \;7.39\;\mu m$.

Considering that cracks may also have initiated at the internal free surface of the capillary, the capillary surface roughness was also measured. Using Electrical Discharge Machining (EDM), the specimens were longitudinally cut halfway through the capillary. The bottom half of the capillary was scanned three times using a scan length of $l_{t}$ = 15 mm and cut-off wavelength of 2.5 mm. Large differences can be noted for the average $R_{a}$ and maximum $R_{t}$ values in between drilled and printed capillaries. The surface finish of drilled capillaries in wrought Ti-6Al-4V (Specimens 4–6) fell between $0.55\;\mu m\;\leq \;R_{a}\;\leq 0.65\;\mu m$ and $5.89\;\mu m\;\leq \;R_{t}\;\leq 10.20\;\mu m$. The printed capillaries in Specimen 9 and Specimen 10 have a more rough surface, characterized by $13.83\;\mu m\;\leq \;R_{a}\;\leq 13.88\;\mu m$ and $70.40\;\mu m\;\leq \;R_{t}\;\leq \;72.80\;\mu m$. The additional drilling operation on the printed capillaries in Specimens 11–13 has not been successful in all specimens. Although the capillary in Specimen 11 obtained a surface finish as good as the drilled capillaries in the wrought Specimens 4–6, some deep notches remained present in Specimens 12 and 13.

The hardness of the specimens was measured using Vickers hardness with a Struers Duramin tester (Struers, Ballerup, Denmark). Prior to the hardness measurements, the surface was subsequently polished with a P1200 and P4000 abrasive grinding paper in order to reduce the surface roughness ($R_{a}\;\leq \;0.1$) as to improve the read-out of the hardness measurements. A 9.807 N load was applied during 10 s for all hardness measurements. Six hardness measurements were performed on each specimen and the average hardness and standard deviation are reported in Table 1. The conventional specimens had a Vickers hardness of approximately 351–356 HV, with Specimen 5 being an outlier with a significantly lower hardness (341 HV). The additively manufactured Specimens 7–13 were all found to be considerably harder (372–403 HV), except for Specimen 9 for which only a little hardness increase was observed (360 HV). Although additively manufactured Specimens 7–10 and Specimens 11–13 were subjected to different heat treatments, no clear differences in hardness could be observed.

## 4. Test Results

Important differences with respect to fatigue initiation location and stress level at failure were found when comparing conventional specimens and additively manufactured specimens, with and without capillaries. A summary of all the specimen’s test results can be found in Table 1, including the fatigue test results as well as the hardness and roughness measurements of the outer surface and capillary surface. A fractographic analysis was performed on all specimens, except for Specimen 5 and Specimen 12 that failed due to the stress concentrations around the rollers. A detailed discussion on the test results is provided in this section.

**Figure 5:**
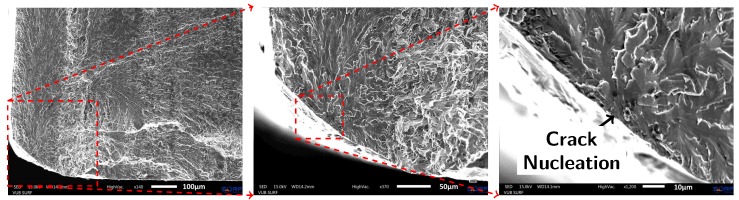
SEM images of the fracture surface of Specimen 1 (wrought material without capillaries). The fatigue crack initiated from the bottom left corner of the specimen.

**Figure 6:**
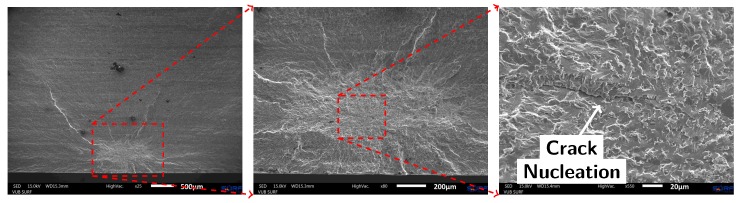
SEM images of the fracture surface of Specimen 3 (wrought material without capillaries). The fatigue crack initiated from an internal defect near the bottom of the specimen.

The three first specimens, manufactured from conventional Ti-6Al-4V, without capillary failed between 820 and 896 MPa. Initially loaded at 589 MPa, the first two specimens survived three steps and failed in the fourth step at a load level of 818 MPa. The third reference specimen survived even the fourth step and had a fatigue strength of 896 MPa. The presented fatigue strengths are in very good correspondence with the literature considering the same test set-up and procedure. Strantza et al. reported three specimens that failed at a stress level of 820 MPa [22]. Figure 5 presents the SEM micrographs taken from the fracture surface of Specimen 1. The fatigue crack nucleations are located at the bottom left corner of the specimen. Similarly, Specimen 2 failed due to an internal defect also located in the beam corner. Specimen 3 failed due to the presence of an internal defect in the bulk material, as shown in Figure 6.

**Figure 7:**
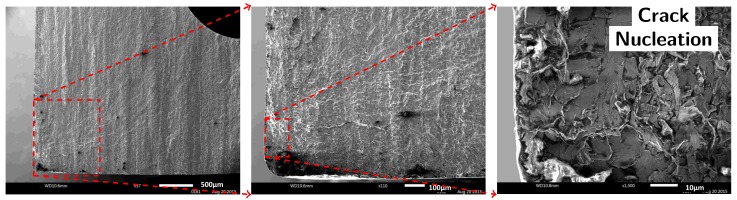
SEM images of the fracture surface of Specimen 4 (wrought material with capillary). The fatigue crack initiated from the bottom left corner of the beam.

Three other specimens, also produced from wrought Ti-6Al-4V, but with integrated capillaries, showed both better and worse fatigue properties than the specimens without capillaries. Specimen 4 failed only at a load level of 976 MPa, while Specimens 5 and 6 failed at 662 MPa. Specimen 5 did not fail in between the inner rollers of the test set-up, but at the contact point with an outer roller, close to the M5 connection with the pressure sensor. The stress concentration around the roller led to premature failure of the specimen. Although having a considerable lower hardness (341 HV) as compared to all other specimens, the actual fatigue strength of the specimen could have been higher than 662 MPa since 459, 360 cycles already survived. In order to overcome the issue of stress concentrations around the rollers, the threaded M5 connections have to be redesigned for future tests in order to maximize the distance between the M5 thread zone and the load insertion by the roller. Fractographic analysis revealed that Specimens 4 and 6 with integrated capillary failed due to a fatigue crack that initiated from the beam corner that was located closest to the capillary. Figure 7 presents the fracture surface of Specimen 4. The fractographic analysis of Specimen 6 is not shown in the current paper since it is very similar to that of Specimen 4. The fatigue crack did not initiate at the capillary surface, likely because of the lower stress levels around the capillary and the low capillary surface roughness ($0.55\;\mu m\;\leq \;R_{a}\;\leq \;0.65\;\mu m$).

**Figure 8:**
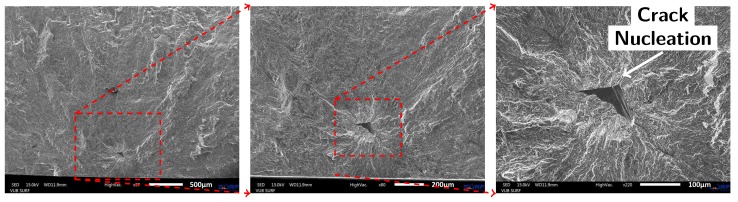
SEM images of the fracture surface of Specimen 7 (AM material without capillary). The fatigue crack initiated from an internal defect near the bottom of the sample.

In contrast with the predictions from the literature, the stress level at failure of the additively manufactured specimen without capillaries were considerably higher than the reference specimens produced from wrought Ti-6Al-4V. Based on previous testing experience on a Ti-6Al-4V specimen produced by laser based DED (but without stress relief and therefore not included in this study), which failed at a load level of 1293 MPa, it was decided to increase the initial load level to reduce testing time. Starting at initial load levels of 896 and 974 MPa, respectively, Specimen 7 and Specimen 8 only failed in the fourth step at a load level of 1208 MPa. As opposed to Specimen 7, Specimen 8 was subjected to a higher initial load level but with smaller load increments, but both specimens failed at the same load level of 1208 MPa. At these load levels, a considerable part of the specimen is already stressed beyond the yield limit of the material. Since further increasing the stress level would not have led to interpretable results, Specimen 8 that survived 500,000 cycles at 1208 MPa was subjected to the same loading until it finally failed. As compared to the reference samples from wrought Ti-6Al-4V, the AM specimens resisted more than two million more cycles at increasing load levels beyond the failure level of the wrought Ti-6Al-4V. These observations are in line with the higher hardness (370–390 HV) of the specimens as compared to the wrought Ti-6Al-4V specimens (340–356 HV), expecting higher tensile strength and improved fatigue performance for the harder specimens. Figure 8 shows the micrographs of the fracture surface of Specimen 7. The rivermarks that are present in the micrograph on the right in Figure 8 indicate that the crack was nucleated due to a triangular shaped void located in the bulk material.

**Figure 9:**
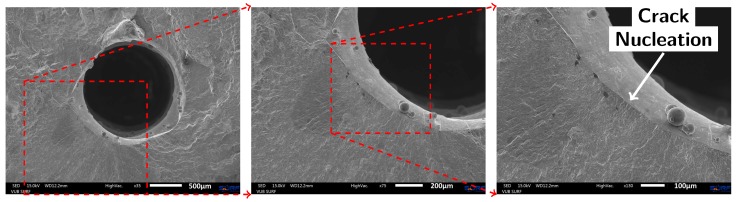
SEM images of the fracture surface of Specimen 9 (AM material with printed capillary) reveal that the fatigue crack initiated from a notch formed by the severe surface roughness at the capillary.

The capillary surface roughness deteriorated the fatigue performance of Specimen 9 and Specimen 10. Fatigue cracks have initiated at the capillary surface at stress levels (589 and 662 MPa) well below those obtained for the additively manufactured specimens without capillaries. The fracture surface of Specimen 9 is shown in Figure 9. In addition, Specimen 10 failed due to a fatigue crack that initiated from the capillary surface. The layerwise addition of contours around the capillary in the vertical build direction has introduced roughness in the capillary surface. The stress concentrations that result thereof lead to premature failure of the specimen. The surface roughness $R_{a}$ of a capillary produced by laser based DED is found to be 10 times higher than that of a drilled capillary.

**Figure 10:**
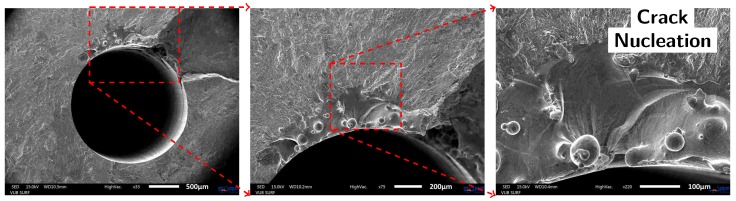
SEM images of the fracture surface of Specimen 13 (AM material with printed capillary after drilling operation). The drilling operation did not sufficiently remove the surface roughness of the printed capillaries and therefore did not solve the initiation issue around the capillary.

Because of the latter conclusions from the fractographic analysis, three more specimens (with initial printed capillaries with a diameter of 1.4–1.8 mm) were drilled afterwards with a drill of 2 mm diameter, in an attempt to reduce the capillary surface roughness. Initially loaded at stress levels similar to previous specimens with printed capillaries, the specimen already failed in the initial load step. The specimen failed at load levels of 433 MPa and 589 MPa. As is clear from the fracture surface of Specimen 13 in Figure 10, the drilling operation did not remove all traces of the additive building process prior to the drilling operation. Fractographic analysis revealed that the remaining “notches” initiated the fatigue crack. Specimen 11 failed due to the presence of a gas inclusion of approximately 200 µm by 500 µm. Specimen 12 failed at the roller contact. The M5-thread connection for the pressure sensor is found to be located too close to the load insertion points (the rollers). New test specimens will be designed differently such that the M5-thread is located in a stress-less zone.

**Figure 11:**
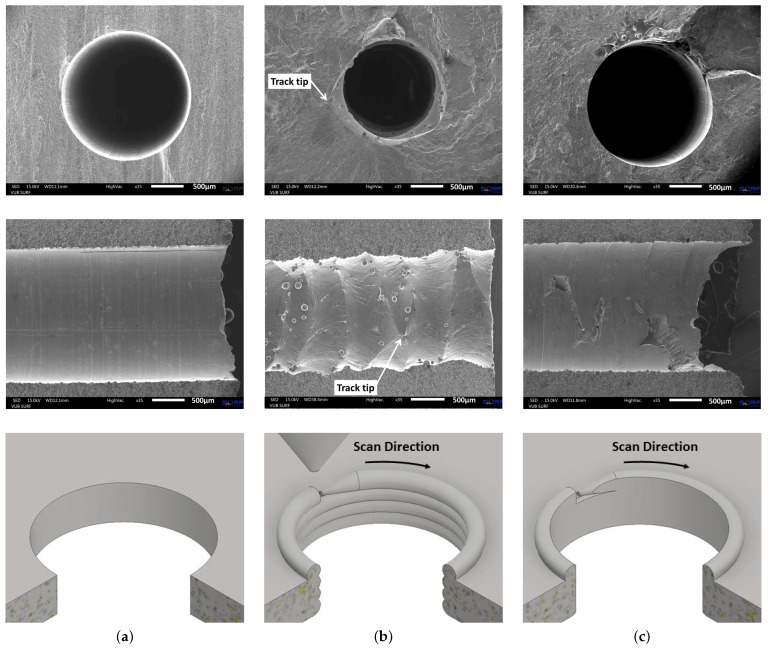
Cross sectional view (top), longitudinal view (middle) and CAD representation of the production process (bottom) of a (**a**) drilled capillary in wrought Ti-6Al-4V (Specimen 6), (**b**) vertically built capillary by laser based DED (Specimen 9) and (**c**) vertically built capillary by laser based DED (Specimen 9) with additional deep gun drilling (Specimen 13). The fracture surface is on the right for the middle figures.

Figure 11 allows comparing the capillary surface finish of a drilled capillary in wrought Ti-6Al-4V material (a), the additively manufactured capillaries using the laser based DED process (b) and the additively manufactured capillaries that were drilled through to reduce the capillary surface roughness (c). Figure 11b,c especially deserve some more attention. As is clear from the middle figure in column (b), the tracks that are clearly visible as vertical lines on the capillary surface, are not all exactly parallel to each other. This is because of the lack of proper process control by the use of continuous laser power (500 W) and constant linear scan speed (1000 mm/min). With the start of each contour around the capillary, the laser is switched on and the nozzle moves at a constant speed. In the very early beginning of the deposition, the material is not sufficiently heated and the melt pool did not grow to its full size before the nozzle moved away, giving rise to a very small track tip. Gradually heating up the substrate, the melt pool reached its intended size and with it also the track became wider. Each such start location of a track is seen as a narrowed track, as indicated on Figure 11b. These locations are regions of larger capillary diameters and are thus locations of deeper notches. Even with the drilling operation, some of these deep notches remained present, as depicted in Figure 11c. A proper closed loop process control system should increase the initial laser power such that the melt pool gains its intended width before moving the nozzle [28,29].

## 5. Simulations

Simulations were performed to better understand the impact of the addition of a capillary feature on the fatigue performance of the specimens. Since considerable differences were recorded in the fatigue response of drilled and additively manufactured capillaries, two distinct cases were simulated: an ideal capillary without roughness modelling (referred to as “Ideal Capillary”) and capillaries with inclusion of the effect of the surface roughness resulting from the AM process (referred to as “Capillary with Roughness Modelling”). The latter modelling case will be based on a cut view of the actual additively manufactured capillaries. Using this methodology, the effect of the addition of a capillary can be decoupled from the effect of the surface finish of the integrated capillary. The impact of the integration of a capillary is derived by comparing of the simulation results of the “Ideal Capillary” with the reference case without capillary. The effect of the capillary surface roughness is analysed through the comparison of the simulated results of an “Ideal Capillary” and the “Capillary with Roughness Modelling”.

All simulations were performed using the ABAQUS FEM modelling software (version 6.14, Dassault Systèmes, Vélizy-Villacoublay, France). The load was inserted to the specimen via a surface pressure load of 1 mm width over the full thickness (12 mm) of the specimen and at the location of the inner rollers 50 mm apart. The outer rollers were also simulated through small surfaces of 1 mm width and the full thickness of the specimen (12 mm) to which displacement in the Y-direction and rotations around Y and Z were restricted. The specimens were virtually loaded with a load of ${}^{37.7}\;/\;{}_{2}$ kN per roller, leading to maximum tensile stress of 589 MPa at the outer surface of the specimen. A tetrahedron mesh was used with a general mesh size of 1 mm and a mesh refinement of 0.25 mm around the simulated notch. The maximum principal stress levels (in MPa) are plotted along the Z-direction (length direction of the specimen) and the X-direction (from the corner with capillary (x = 0 mm) to the other corner (x = 12 mm)).

### 5.1. Integration of an Ideal Capillary

The modelling of an “Ideal Capillary” allows analysing the base effect of the addition of a capillary, without its surface finishing complications and resembles quite well the case of a drilled capillary in the conventional Ti-6Al-4V specimens.

**Figure 12:**
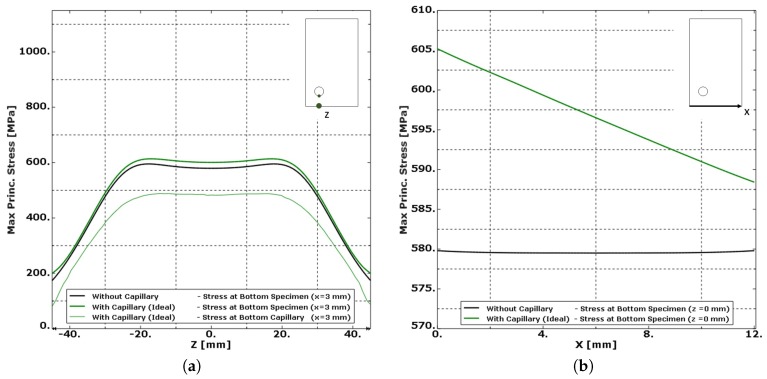
The maximum principal stress level plotted in function of (**a**) the longitudinal Z-direction at the bottom of the specimen and bottom of the capillary and (**b**) the X-direction at the bottom of specimen. The integration of a capillary slightly increases (+3%) the overall stress level in the specimen. The stress level at the capillary is significantly lower than that at the bottom of the specimen. The stress level increases linearly by 3% from the corner without capillary to the corner with capillary.

A comparison of the modelling results presented in Figure 12 shows the effect of the integration of a capillary. According to Figure 12a, the addition of the capillary slightly increases (+3%) all stress levels inside the specimen. The stress levels at the capillary remain well below the stress levels found at the bottom of the specimen. It is therefore expected that for materials without defects, fatigue cracks will not initiate at the capillary location. Fractographic analysis of specimens with drilled capillaries in wrought Ti-6Al-4V indeed revealed that the fatigue cracks did not initiate at the capillary surface.

When considering the stress pattern along the X-direction in Figure 12b, the proximity of the capillary near one corner slightly increases the stress level in that corner. Although the difference of the stress level from one corner to the other is limited to only 3%, it is possibly the reason why fatigue cracks seem to prefer this corner as fatigue initiation location as found during the fractographic analysis of Specimens 4 and 6. The addition of a capillary could alter the fatigue performance of the component; however, further studies will be needed to analyse this effect.

### 5.2. Integration of a Capillary with Roughness Modelling

**Figure 13:**
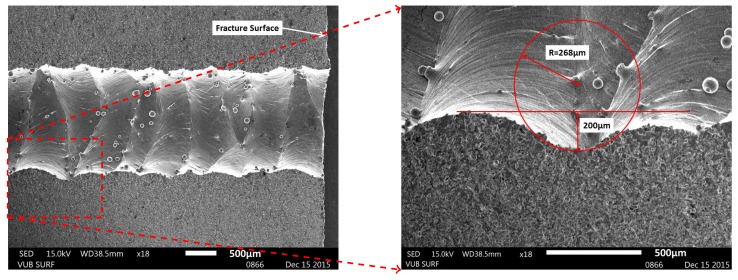
Longitudinal cut view of the surface roughness of the printed capillary of Specimen 9: (**a**) a general overview and (**b**) a detailed view with derived dimensions as input for the simulations.

Although the integration of an “Ideal Capillary” did not alter the fatigue initiation location, the roughness of the additively manufactured capillary leads to fatigue initiation at the capillary surface. In an attempt to estimate the severity of the stress concentrations introduced by the surface roughness, simulations were performed. The geometry of the simulated “notch” was derived from a longitudinal cut view of Specimen 9, provided in Figure 13. Only one notch is considered in the simulations and is modelled as a torus with a minor radius of 268 µm and a major radius of 932 µm such that the indentation with respect to the minimal capillary diameter of 2 mm equals 200 µm. The notch is located in the middle of the beam, between the inner rollers where tensional stresses are the highest. The same load of ${}^{37.7}\;/\;{}_{2}$ kN per roller, the load level at which Specimen 9 failed, was again applied to the specimen.

**Figure 14:**
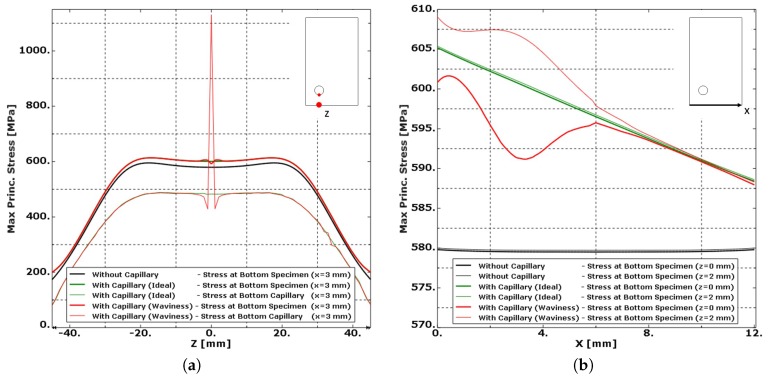
The stress level is plotted in a function of (**a**) the longitudinal Z-direction at the bottom of the specimen and bottom of the capillary and (**b**) at the bottom of the specimen along the X-direction for z = 0 mm and z = 2 mm. The stress concentration caused by the roughness of the capillary surpasses all stress levels of the specimen with smooth capillaries. The capillary becomes the place were fatigue initiation can be expected.

It is clear from the simulation results presented in Figure 14 that the surface roughness of the laser based DED process induced stress concentrations that surpass all stress levels expected in a specimen with an “Ideal Capillary”. The roughness on the capillary makes the capillary surface a preferred location for crack initiation. On top of this capillary roughness, the presence of unmelted particles furthermore increased the capillary surface roughness and can cause crack nucleation. Figure 14b presents the results of the same simulations along the X-direction at the outer surface of the specimen, where only small influences are noted. The highest stress levels at the outer surface of the specimen are not found just below the “notch” (z = 0 mm), but at z = 2 mm.

## 6. Conclusions

Material properties of AM Ti-6Al-4V material are widely reported in the literature for different AM processes. Showing a significant spread in fatigue performances, most of the AM Ti-6Al-4V material performs weaker than wrought Ti-6Al-4V. A new SHM methology for AM components was presented to detect fatigue cracks. A capillary is to be integrated in the component by means of AM techniques and pressurized afterwards. The presence of a fatigue crack is derived on the basis of the pressure monitoring of the embedded capillary. Fatigue cracks initiating at the specimen’s surface propogate through the material and reach the capillary. By breaching through the capillary, the internal capillary pressure equalizes the ambient pressure by the leak flow passing through the crack. The integration of such a capillary feature may not jeopardize the structural integrity of the component. Previous fatigue tests on a laser based PBF Ti-6Al-4V specimen with an integrated capillary have shown that the capillary feature did not lead to fatigue initiation at the capillary surface [22]. As an extension, this work analyses the effect of the integration of a capillary feature to Ti-6Al-4V specimens produced by laser based DED.

Four point bending fatigue tests were performed on Ti-6Al-4V specimen produced from wrought plate material and AM material produced by laser based DED. Specimens with and without integrated capillaries are tested. The step method was used to estimate the fatigue strength of the specimen, and fractographic analysis was used to retrieve the fatigue initiation location. Hardness and surface roughness measurements were performed to better understand the fatigue test results. Simulations were confirmed the findings from the experimental test campaign.

Considering the wrought Ti-6Al-4V material, it was found that the addition of a drilled capillary did not alter the fatigue initiation location. Cracks still initiated at the outer surface of the specimen. Simulations confirmed that the stress levels expected around a capillary with the absence of roughness are expected to be significantly lower than at the outer surface of the specimen where tensional stresses were the highest. Fatigue cracks are therefore not expected to initiate at the capillary surface. The two tested specimens failed due to a fatigue crack that initiated from the specimen corner closest to the capillary. The simulations also revealed that the stress level in this corner is 3% higher as compared to the corner further away from the capillary. As only two specimens were tested and the differences in stress levels are limited, these findings have to be further verified in a dedicated test program.

The laser based DED specimens without capillaries failed at a higher load level than the wrought Ti-6Al-4V material. These findings are in line with the hardness increase observed. However, the addition of a printed capillary deteriorated the fatigue performance. Fractographic analysis revealed that all fatigue cracks initiated from the capillary surface as a result of the roughness introduced by the laser based DED process. Simulations confirmed that the expected stress concentrations at the capillary surface surpass all stress levels normally expected in a specimen with an “Ideal Capillary” without capillary roughness. It is thus expected and confirmed by experiments that the fatigue cracks initiated at the capillary surface. The attempt to reduce the surface roughness by deep gun drilling through the capillary did not solve the problem, as some “notches” were not removed and became the crack initiation site. Because of the large impact of the capillary surface roughness, no interpretations can be made on the use of a different thermal treatment, nor whether the corner closer to the capillary is in favour for crack initiation.

From this investigation, it is concluded that the integration of a capillary does not significantly change the fatigue properties of a specimen. The eSHM principle therefore does not jeopardize the structural integrity of the structure. However, the laser based DED process currently lacks a proper capillary surface finish. The roughness that resulted from the layerwise addition of contours around the capillary limited the fatigue performance of the specimens with printed capillaries. A first attempt to reduce the surface roughness by deep hole drilling through the existing additive manufactured holes did not work out properly since notches remained present after deep gun drilling. Future work will be oriented towards drilling holes in AM specimens and subsequently in investigating a proper AM process for capillaries with a smooth surface finish. Surface quality of the integrated capillary is of primary importance to not influence the structural integrity of the component to be monitored.

## Figures and Tables

**Table 1:** Summary of Ti-6Al-4V specimens of four-point bending fatigue tests, hardness and roughness measurements. Standard deviation (STD) is given in between round brackets on the right of the average (AVG) value: AVG. (STD.).

	Total	Stress Level		Cycles	Fatigue Initiation		Hardness		External Roughness				Capillary Roughness			
	Steps	Initial	Failure]	Last Step	Location	Cause	Vickers		$R_{a}$		$R_{t}$		$R_{a}$		$R_{t}$	
	(–)	(MPa)	(MPa)	(–)	(–)	(–)	(HV)	(HV)	(µm)	(µm)	(µm)	(µm)	(µm)	(µm)	(µm)	(µm)
**Wrought Ti-6Al-4V Specimen without Capillary**																
**Specimen 1**	4	589	818	149,429	Corner	Roughness	353.00	(16.07)	0.51	(0.13)	7.39	(2.28)	-	-	-	-
**Specimen 2**	4	589	818	78,851	Corner	Defect	352.00	(8.22)	0.25	(0.05)	2.59	(0.40)	-	-	-	-
**Specimen 3**	5	589	896	247,865	Bulk	Defect	351.50	(9.33)	0.35	(0.07)	3.90	(0.79)	-	-	-	-
**Wrought Ti-6Al-4V Specimen with Drilled Capillary**																
**Specimen 4**	6	589	974	384,270	Corner	Roughness	356.17	(20.05)	0.24	(0.06)	2.66	(0.42)	0.65	(0.17)	10.20	(1.82)
**Specimen 5**	2	589	662	459,360	Roller	Roller	341.33	(20.65)	0.40	(0.50)	3.23	(0.42)	0.62	(0.10)	8.35	(1.25)
**Specimen 6**	4	433	662	364,287	Corner	Roughness	352.67	(18.95)	0.52	(0.10)	5.12	(1.20)	0.55	(0.04)	5.86	(0.66)
**Laser Based DED Ti-6Al-4V Specimen without Capillary**																
**Specimen 7**	4	896	1208	308,275	Bulk	Defect	388.67	(5.09)	0.36	(0.11)	3.79	(0.59)	-	-	-	-
**Specimen 8**	4	974	1208	951,150	Bulk	Defect	372.50	(6.83)	0.26	(0.12)	3.15	(0.81)	-	-	-	-
**Laser Based DED Ti-6Al-4V Specimen with Additive Manufactured Capillary**																
**Specimen 9**	3	433	589	234,025	Capillary	Roughness	359.67	(9.09)	0.28	(0.18)	4.89	(1.55)	13.88	(0.46)	70.40	(5.21)
**Specimen 10**	3	511	662	262,773	Capillary	Roughness	376.67	(2.73)	0.32	(0.20)	7.18	(2.66)	13.83	(2.20)	72.80	(12.35)
**Laser Based DED Ti-6Al-4V Specimen with Drilled Capillary**																
**Specimen 11**	1	433	433	134,413	Edge	Defect	372.83	(11.03)	0.79	(0.11)	5.82	(0.31)	0.60	(0.00)	7.10	(0.25)
**Specimen 12**	1	433	433	236,609	Roller	Roller	383.17	(4.54)	0.38	(0.12)	3.55	(0.57)	1.63	(0.06)	57.00	(3.85)
**Specimen 13**	1	589	589	219,377	Capillary	Roughness	403.75	(10.54)	0.43	(0.10)	4.12	(0.71)	7.53	(0.64)	107.30	(17.49)

## Abbreviations

The following abbreviations are used in this manuscript:

AM	Additive Manufacturing
HCF	High Cycle Fatigue
LCF	Low Cycle Fatigue
PBF	Powder Bed Fusion
SLM	Selective Laser Melting
EBM	Electron Beam Melting
DED	Direct Energy Deposition
LMD	Laser Metal Deposition
LENS	Laser Engineered Net Shaping
SHM	Structural Health Monitoring
eSHM	effective Structural Health Monitoring
NDT	Non Destructive Testing
TRL	Technology Readiness Level
SEM	Scanning Electron Microscopy
EDM	Electrical Discharge Machining
AVG	Average
STD	Standard Deviation
